# Olivine-type NaCd(AsO_4_)

**DOI:** 10.1107/S1600536813027256

**Published:** 2013-10-05

**Authors:** Matthias Weil

**Affiliations:** a Institute for Chemical Technologies and Analytics, Division of Structural Chemistry, Vienna University of Technology, Getreidemarkt 9/164-SC, A-1060 Vienna, Austria

## Abstract

The title compound, sodium cadmium orthoarsenate, adopts the olivine [Mg_2_(SiO_4_)] structure type in space group *Pnma*, with Na (site symmetry -1) and Cd (.*m*.) replacing the two Mg positions, and the AsO_4_ tetra­hedron (.*m*.) the SiO_4_ tetra­hedron. The crystal structure is made up of a nearly hexa­gonal closed-packed arrangement of O atoms stacked along [001]. The Na and Cd atoms occupy one half of the octa­hedral voids in alternate layers stacked along [100], and one eighth of the tetra­hedral voids are occupied by As atoms.

## Related literature

For a review of the crystal chemistry of olivines, see: Brown (1982 ▶). For the isotypic phosphate analogue, see: Ivanov *et al.* (1974 ▶); Hata *et al.* (1979 ▶). For other phases in the system Na–Cd–P–O, see: Murashova & Chudinova (1997 ▶); Bennazha *et al.* (2000 ▶); Ben Amara *et al.* (1979 ▶). For standardization of structure data, see: Gelato & Parthé (1987 ▶).

## Experimental

### 

#### Crystal data

NaCd(AsO_4_)
*M* *_r_* = 274.32
Orthorhombic, 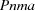

*a* = 11.1585 (17) Å
*b* = 6.550 (1) Å
*c* = 5.2330 (8) Å
*V* = 382.47 (10) Å^3^
*Z* = 4
Mo *K*α radiation
μ = 14.27 mm^−1^
*T* = 293 K
0.06 × 0.04 × 0.04 mm

#### Data collection

Bruker SMART CCD diffractometer
Absorption correction: multi-scan (*SADABS*; Bruker, 2005 ▶) *T*_min_ = 0.481, *T*_max_ = 0.599
4252 measured reflections
656 independent reflections
615 reflections with *I* > 2σ(*I*)
*R*_int_ = 0.030

#### Refinement

*R*[*F* ^2^ > 2σ(*F* ^2^)] = 0.017
*wR*(*F* ^2^) = 0.043
*S* = 1.10
656 reflections
40 parameters
Δρ_max_ = 0.67 e Å^−3^
Δρ_min_ = −0.75 e Å^−3^

### 

Data collection: *SMART* (Bruker, 2005 ▶); cell refinement: *SAINT* (Bruker, 2005 ▶); data reduction: *SAINT*; program(s) used to solve structure: *SHELXS97* (Sheldrick, 2008 ▶); program(s) used to refine structure: *SHELXL97* (Sheldrick, 2008 ▶); molecular graphics: *ATOMS for Windows* (Dowty, 2006 ▶); software used to prepare material for publication: *publCIF* (Westrip, 2010 ▶).

## Supplementary Material

Crystal structure: contains datablock(s) I, global. DOI: 10.1107/S1600536813027256/pj2006sup1.cif

Structure factors: contains datablock(s) I. DOI: 10.1107/S1600536813027256/pj2006Isup2.hkl

Additional supplementary materials:  crystallographic information; 3D view; checkCIF report

## Figures and Tables

**Table 1:** Selected bond lengths (Å)

Cd—O2	2.229 (3)
Cd—O1^i^	2.2629 (18)
Cd—O1^ii^	2.2629 (18)
Cd—O3	2.321 (2)
Cd—O1	2.4030 (18)
Cd—O1^iii^	2.4030 (18)
Na—O2	2.3779 (19)
Na—O2^iv^	2.3779 (19)
Na—O3^v^	2.3814 (18)
Na—O3^vi^	2.3814 (18)
Na—O1^v^	2.5031 (18)
Na—O1^vi^	2.5031 (18)
As—O3^vii^	1.677 (2)
As—O2^viii^	1.687 (2)
As—O1^iii^	1.7030 (17)
As—O1	1.7030 (17)

Symmetry codes: (i) 
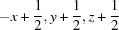
; (ii) 
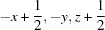
; (iii) 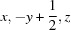; (iv) 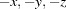; (v) 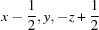; (vi) 
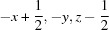
; (vii) 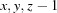; (viii) 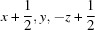.

**Fig. 1.**
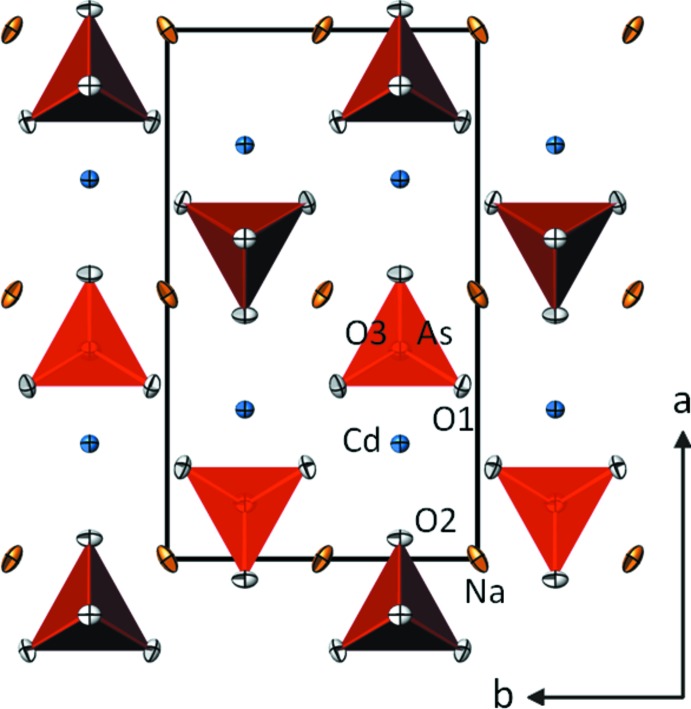
The olivine-type crystal structure of NaCd(AsO4) in a projection along [001]. Displacement ellipsoids are drawn at the 74% probability level.

## supplementary crystallographic information

### 1. Comment

Up to now there are no reports on crystal structure determinations of phases in the system Na–Cd–As–O, whereas for the analogous system Na–Cd–P–O several phases with compositions NaCd(PO_4_) (Ivanov *et al.*, 1974; Hata *et al.*, 1979), NaCd(PO_3_)_3_ (Murashova & Chudinova, 1997), Na_2_Cd_3_(P_2_O_7_)_2_ (Bennazha *et al.*, 2000) and Cd_4_Na(PO_4_)_3_ (Ben Amara *et al.*, 1979) have been structurally fully characterized. In this article the synthesis and crystal structure of NaCd(AsO_4_) is reported.

NaCd(AsO_4_) crystallizes in the olivine [Mg_2_(SiO_4_)] structure type. A review on the crystal chemistry of olivines and spinels has been published some time ago by Brown (1982). The olivine structure is characterized by an almost hexagonal closed-packed arrangement of oxygen atoms parallel to (001) with a stacking sequence *ABAB* along [001] with one half of the octahedral voids occupied by magnesium sites, and one-eighth of the tetrahedral voids by silicon sites. In the structure of NaCd(AsO_4_), the two magnesium sites and the silicon site are replaced by unique sodium and cadmium sites and an arsenic site, respectively. The resulting NaO_6_ octahedron has 1 symmetry, and the CdO_6_ octahedron and the AsO_4_ tetrahedron both have .*m*. symmetry.

In comparison with the isotypic structure of NaCd(PO_4_) (Ivanov *et al.*, 1974), the Cd—O and Na—O distances are virtually the same, with mean bond lengths for the CdO_6_ and NaO_6_ octahedra of 2.307 (71) Å and 2.398 (54) Å in the phosphorus compound, and of 2.314 (75) Å and 2.421 (64) Å, respectively, in the arsenic compound. As expected, only the distances within the PO_4_ (mean P—O distance 1.541 (12) Å) and AsO_4_ (1.693 (13) Å) tetrahedra differ, because of the different sizes of P(V) and As(V).

### 2. Experimental

To an aqueous 0.05 *M* solution (20 ml) of cadmium acetate, an aqueous solution of 0.05 *M* Na_2_HAsO_4_ (20 ml) was added. Together with the mother liquor, parts of the colourless precipitate were transferred to a 10 ml Teflon insert (filling degree one-half). The insert was then sealed and heated in a steel autoclave under autogenous pressure at 338 K for ten days. Few colourless single crystals of the title compound with a block-like form were obtained after the reaction time, and were manually separated from the reaction mixture that was not further examined.

### 3. Refinement

Atomic coordinates of the isotypic NaCd(PO_4_) (Ivanov *et al.*, 1974) in space group *Pnma* were taken as starting parameters for refinement of NaCd(AsO_4_). Besides a structure refinement of NaCd(PO_4_) in *Pnma*, also refinements of this structure in the non-centrosymmetric space group No. 33 were performed with settings in *Pn*2_1_*a* (Ivanov *et al.*, 1974) and *P*2_1_*nb* (Hata *et al.*, 1979). However, adopting these non-centrosymmetric structure models for refinement of NaCd(AsO_4_) converged with significantly higher residual parameters and with large correlation matrix elements. Therefore the structure model in space group *Pnma* was considered as being correct, in agreement with other olivine-type structures. Structure data were finally standardized with *STRUCTURE TIDY* (Gelato & Parthé, 1987).

### Crystal data

**Table:**

NaCd(AsO_4_)	*F*(000) = 496
*M**_r_* = 274.32	*D*_x_ = 4.764 Mg m^−^^3^
Orthorhombic, *P**n**m**a*	Mo *K*α radiation, λ = 0.71073 Å
Hall symbol: -P 2ac 2n	Cell parameters from 2012 reflections
*a* = 11.1585 (17) Å	θ = 3.7–30.9°
*b* = 6.550 (1) Å	µ = 14.27 mm^−^^1^
*c* = 5.2330 (8) Å	*T* = 293 K
*V* = 382.47 (10) Å^3^	Block, colourless
*Z* = 4	0.06 × 0.04 × 0.04 mm

### Data collection

**Table:**

Bruker SMART CCD diffractometer	656 independent reflections
Radiation source: fine-focus sealed tube	615 reflections with *I* > 2σ(*I*)
Graphite monochromator	*R*_int_ = 0.030
ω scans	θ_max_ = 31.0°, θ_min_ = 3.7°
Absorption correction: multi-scan (*SADABS*; Bruker, 2005)	*h* = −16→15
*T*_min_ = 0.481, *T*_max_ = 0.599	*k* = −8→9
4252 measured reflections	*l* = −7→7

### Refinement

**Table:**

Refinement on *F*^2^	0 restraints
Least-squares matrix: full	Primary atom site location: structure-invariant direct methods
*R*[*F*^2^ > 2σ(*F*^2^)] = 0.017	Secondary atom site location: difference Fourier map
*wR*(*F*^2^) = 0.043	*w* = 1/[σ^2^(*F*_o_^2^) + (0.0225*P*)^2^ + 0.1997*P*] where *P* = (*F*_o_^2^ + 2*F*_c_^2^)/3
*S* = 1.10	(Δ/σ)_max_ < 0.001
656 reflections	Δρ_max_ = 0.67 e Å^−^^3^
40 parameters	Δρ_min_ = −0.75 e Å^−^^3^

### Special details

**Table:**

Geometry. All e.s.d.'s (except the e.s.d. in the dihedral angle between two l.s. planes) are estimated using the full covariance matrix. The cell e.s.d.'s are taken into account individually in the estimation of e.s.d.'s in distances, angles and torsion angles; correlations between e.s.d.'s in cell parameters are only used when they are defined by crystal symmetry. An approximate (isotropic) treatment of cell e.s.d.'s is used for estimating e.s.d.'s involving l.s. planes.
Refinement. Refinement of *F*^2^ against ALL reflections. The weighted *R*-factor *wR* and goodness of fit *S* are based on *F*^2^, conventional *R*-factors *R* are based on *F*, with *F* set to zero for negative *F*^2^. The threshold expression of *F*^2^ > σ(*F*^2^) is used only for calculating *R*-factors(gt) *etc*. and is not relevant to the choice of reflections for refinement. *R*-factors based on *F*^2^ are statistically about twice as large as those based on *F*, and *R*- factors based on ALL data will be even larger.

### Fractional atomic coordinates and isotropic or equivalent isotropic displacement parameters (Å^2^)

**Table:**

	*x*	*y*	*z*	*U*_iso_*/*U*_eq_	
Cd	0.21753 (2)	0.2500	0.50818 (5)	0.00974 (8)	
Na	0.0000	0.0000	0.0000	0.0140 (3)	
As	0.39805 (3)	0.2500	0.06207 (6)	0.00795 (9)	
O1	0.32677 (15)	0.0493 (3)	0.2024 (3)	0.0122 (3)	
O2	0.0386 (2)	0.2500	0.3191 (5)	0.0127 (5)	
O3	0.3944 (2)	0.2500	0.7417 (5)	0.0116 (5)	

### Atomic displacement parameters (Å^2^)

**Table:**

	*U*^11^	*U*^22^	*U*^33^	*U*^12^	*U*^13^	*U*^23^
Cd	0.00894 (13)	0.00954 (14)	0.01073 (13)	0.000	0.00028 (7)	0.000
Na	0.0209 (7)	0.0128 (8)	0.0084 (7)	0.0086 (6)	−0.0005 (5)	−0.0027 (5)
As	0.00785 (16)	0.00855 (18)	0.00743 (16)	0.000	0.00039 (11)	0.000
O1	0.0157 (8)	0.0086 (8)	0.0124 (8)	−0.0029 (6)	0.0025 (6)	0.0004 (6)
O2	0.0088 (10)	0.0188 (14)	0.0106 (11)	0.000	0.0015 (8)	0.000
O3	0.0119 (11)	0.0158 (13)	0.0069 (10)	0.000	0.0003 (8)	0.000

### Geometric parameters (Å, º)

**Table:**

Cd—O2	2.229 (3)	Na—O3^v^	2.3814 (18)
Cd—O1^i^	2.2629 (18)	Na—O3^vi^	2.3814 (18)
Cd—O1^ii^	2.2629 (18)	Na—O1^v^	2.5031 (18)
Cd—O3	2.321 (2)	Na—O1^vi^	2.5031 (18)
Cd—O1	2.4030 (18)	As—O3^vii^	1.677 (2)
Cd—O1^iii^	2.4030 (18)	As—O2^viii^	1.687 (2)
Na—O2	2.3779 (19)	As—O1^iii^	1.7030 (17)
Na—O2^iv^	2.3779 (19)	As—O1	1.7030 (17)
			
O2—Cd—O1^i^	90.21 (5)	O3^v^—Na—O1^vi^	98.07 (7)
O2—Cd—O1^ii^	90.21 (5)	O3^vi^—Na—O1^vi^	81.93 (7)
O1^i^—Cd—O1^ii^	120.07 (9)	O1^v^—Na—O1^vi^	180.00 (5)
O2—Cd—O3	174.59 (9)	O3^vii^—As—O2^viii^	113.04 (12)
O1^i^—Cd—O3	87.09 (5)	O3^vii^—As—O1^iii^	114.82 (8)
O1^ii^—Cd—O3	87.09 (5)	O2^viii^—As—O1^iii^	105.97 (8)
O2—Cd—O1	99.14 (7)	O3^vii^—As—O1	114.82 (8)
O1^i^—Cd—O1	152.11 (7)	O2^viii^—As—O1	105.97 (8)
O1^ii^—Cd—O1	86.33 (4)	O1^iii^—As—O1	101.06 (12)
O3—Cd—O1	85.37 (7)	As—O1—Cd^vi^	125.24 (9)
O2—Cd—O1^iii^	99.14 (7)	As—O1—Cd	95.84 (8)
O1^i^—Cd—O1^iii^	86.33 (4)	Cd^vi^—O1—Cd	131.47 (7)
O1^ii^—Cd—O1^iii^	152.11 (7)	As—O1—Na^ii^	90.41 (7)
O3—Cd—O1^iii^	85.37 (7)	Cd^vi^—O1—Na^ii^	109.66 (7)
O1—Cd—O1^iii^	66.33 (8)	Cd—O1—Na^ii^	92.76 (6)
O2—Na—O2^iv^	180.00 (9)	As^v^—O2—Cd	132.00 (14)
O2—Na—O3^v^	89.37 (6)	As^v^—O2—Na	95.20 (9)
O2^iv^—Na—O3^v^	90.63 (6)	Cd—O2—Na	118.29 (8)
O2—Na—O3^vi^	90.63 (6)	As^v^—O2—Na^ix^	95.20 (9)
O2^iv^—Na—O3^vi^	89.37 (6)	Cd—O2—Na^ix^	118.29 (8)
O3^v^—Na—O3^vi^	180.00 (10)	Na—O2—Na^ix^	87.04 (9)
O2—Na—O1^v^	67.30 (7)	As^x^—O3—Cd	123.17 (13)
O2^iv^—Na—O1^v^	112.70 (7)	As^x^—O3—Na^ii^	121.26 (9)
O3^v^—Na—O1^v^	81.93 (7)	Cd—O3—Na^ii^	98.11 (8)
O3^vi^—Na—O1^v^	98.07 (7)	As^x^—O3—Na^xi^	121.26 (9)
O2—Na—O1^vi^	112.70 (7)	Cd—O3—Na^xi^	98.11 (8)
O2^iv^—Na—O1^vi^	67.30 (7)	Na^ii^—O3—Na^xi^	86.89 (8)

Symmetry codes: (i) −*x*+1/2, *y*+1/2, *z*+1/2; (ii) −*x*+1/2, −*y*, *z*+1/2; (iii) *x*, −*y*+1/2, *z*; (iv) −*x*, −*y*, −*z*; (v) *x*−1/2, *y*, −*z*+1/2; (vi) −*x*+1/2, −*y*, *z*−1/2; (vii) *x*, *y*, *z*−1; (viii) *x*+1/2, *y*, −*z*+1/2; (ix) −*x*, *y*+1/2, −*z*; (x) *x*, *y*, *z*+1; (xi) *x*+1/2, −*y*+1/2, −*z*+1/2.
